# LncRNA ALMS1‐IT1 modulates ferroptosis and immune evasion in colorectal cancer through activating STAT3

**DOI:** 10.1111/jcmm.70103

**Published:** 2024-09-27

**Authors:** Zhaoying Wu, Junwei Zou, Hao Xie, Jie Wang, Yong Huang, Fei Liu, Chungen Xing

**Affiliations:** ^1^ Department of Gastrointestinal Surgery The Second Affiliated Hospital of Wannan Medical College Wuhu Anhui China; ^2^ Department of Gastrointestinal Surgery The Second Affiliated Hospital of Soochow University Suzhou Jiangsu China; ^3^ Department of Gastroenterology The Second Affiliated Hospital of Soochow University Suzhou Jiangsu China

**Keywords:** ALMS1‐IT1, colorectal cancer, ferroptosis, immune evasion, STAT3

## Abstract

Colorectal cancer (CRC) represents a significant malignancy within the digestive system, characterized by high incidence and mortality rates. In recent years, molecular targeted therapy has been introduced as a supplementary strategy in CRC management, complementing traditional modalities such as surgery, radiation and chemotherapy. The identification of novel therapeutic targets for CRC remains critically important. Ferroptosis, a unique form of programmed cell death distinct from apoptosis and necrosis, is characterized by cellular damage resulting from iron‐induced lipid peroxidation, leading to cell death. This study utilizes a combination of bioinformatics analysis and clinical specimen validation to demonstrate that the long non‐coding RNA (lncRNA) ALMS1‐IT1 is significantly upregulated in CRC tissues and strongly associated with ferroptosis. Through a series of experimental investigations, we have determined that ALMS1‐IT1 negatively regulates ferroptosis in CRC cells, thereby promoting cancer growth and metastasis, acting as an oncogenic factor. Furthermore, we explored the molecular interactions of ALMS1‐IT1, revealing its role in activating STAT3 protein phosphorylation. This activation enhances the immune evasion capabilities of CRC cells. Rescue experiments indicated that STAT3 activation is essential for ALMS1‐IT1's suppression of ferroptosis, immune evasion and oncogenic behaviour in CRC. Our findings underscore the critical biological role of ALMS1‐IT1 in the progression of CRC and suggest its potential as a target for drug development.

## 1 INTRODUCTION

Colorectal cancer (CRC) ranks as the fourth most prevalent malignancy globally and the third leading cause of cancer‐related mortality.^1^ It represents a multifaceted disease influenced by genetic, hereditary, environmental and lifestyle factors.^2^ Due to the inadequacy of sensitive biomarkers and efficacious screening modalities, a considerable portion of CRC patients receive diagnoses at advanced stages, leading to dismal 5‐year survival rates.^3^ Despite advancements in diagnosis and therapy, patient prognosis remains unfavourable due to recurrence, metastasis and drug resistance.^4^ Consequently, there is an imperative to explore novel biomarkers and gain deeper insights into the mechanisms underlying CRC to develop improved treatment strategies.

lncRNAs constitute a diverse group of transcripts exceeding 200 nucleotides (nt) in length, characterized by minimal or absent protein‐coding capacity.^5^ Indeed, lncRNAs exhibit versatile interactions with RNA, DNA, and proteins, forming intricate complexes such as RNA–RNA, RNA–DNA, and RNA‐protein. Through these interactions, lncRNAs exert regulatory control over gene expression via diverse mechanisms, including transcriptional modulation, regulation of mRNA stability, and influence on translation processes.^6^, ^7^ Acting as guides, scaffolds, or decoy molecules, lncRNAs can recruit proteins or RNAs to specific genomic loci. Additionally, lncRNAs have the capacity to influence chromatin structure, thereby modulating gene expression patterns.^8^ LncRNAs represent significant constituents of the noncoding RNA category and play pivotal roles in either promoting or suppressing tumour progression.^9^ Certain lncRNAs have been identified to be closely linked with ferroptosis and the prognosis of CRC.^10^, ^11^ Thereafter, lncRNAs are promising direction for investigating putative therapeutic target and drug design strategy for CRC.

Ferroptosis, classified as a regulated cell death (RCD) mechanism,^12^ is instigated by the buildup of lipid peroxides within cells. This process is intricately linked to the maintenance of iron homeostasis, lipid metabolism, and amino acid metabolism.^13^ Given that ferroptosis ensues from iron‐mediated oxidative stress, cancer cells exhibit a heightened susceptibility to ferroptosis compared to normal cells owing to their elevated metabolic demands and reactive oxygen species (ROS) burden. Consequently, tumour cells typically necessitate elevated levels of iron and amino acids to sustain their proliferative capacity.^14^, ^15^ This heightened vulnerability of cancer cells to ferroptosis has spurred investigations into ferroptosis induction as a promising therapeutic strategy, particularly for chemoresistant tumour cells. Recent research has highlighted the potential of a novel ferroptosis‐related gene signature as a promising biomarker for predicting the prognosis of patients with CRC.^16^, ^17^ Nevertheless, the precise molecular mechanisms underlying the regulation of ferroptosis in CRC have yet to be fully elucidated.

In this study, we investigated the relationship between CRC and ferroptosis as mediated by long non‐coding RNAs (lncRNAs). Through comprehensive bioinformatics analysis, we identified several ferroptosis‐related lncRNAs that are dysregulated in CRC. Among these, the lncRNA ALMS1 intronic transcript 1 (ALMS1‐IT1) was notably upregulated in CRC tissues, a finding that was confirmed using clinical human samples and shown to be strongly correlated with poor patient outcomes. Further investigations demonstrated that ALMS1‐IT1 plays a suppressive role in ferroptosis within CRC cells. Functional assays revealed the oncogenic nature of ALMS1‐IT1 in CRC progression, as it promotes cell proliferation, migration and invasion. Subsequent mechanistic explorations illuminated the molecular pathways influenced by ALMS1‐IT1. Specifically, ALMS1‐IT1 was found to interact with and activate STAT3 by promoting its phosphorylation. Additionally, ALMS1‐IT1 was implicated in enhancing immune evasion in CRC cells through STAT3 activation. Notably, the activation of STAT3 was essential for mediating the effects of ALMS1‐IT1 on both ferroptosis suppression and tumorigenesis in CRC cells. These findings provide valuable insights into potential therapeutic targets for CRC treatment, emphasizing the significance of ALMS1‐IT1 and STAT3 in regulating ferroptosis and tumorigenesis in CRC.

## 2 MATERIALS AND METHODS

### 2.1 Data collection and analysis

The transcriptome data were acquired from CRC patients in The Cancer Genome Atlas (TCGA) database (https://portal.gdc.cancer.gov/). Using the limma R software package (https://www.r‐project.org/), we conducted differential expression analysis to identify ferroptosis‐related genes and lncRNAs that were differentially expressed between tumour and normal samples. The criteria used for screening were |log2(fold change)| >1 and a *p* < 0.05 for ferroptosis‐related gene differences. Subsequently, we employed univariate Cox regression analysis to identify prognosis‐related ferroptosis‐related lncRNAs. The risk score for each sample was calculated through LASSO Cox regression analysis using the expression levels of ferroptosis‐related lncRNAs.

### 2.2 Clinical sample collection

A total of 24 CRC tissue samples, along with corresponding normal adjacent tissues, were collected from patients diagnosed with CRC who underwent surgical resection at The Second Affiliated Hospital of Wannan Medical College. All tissue samples were preserved at −80°C until further analysis. The patients were diagnosed through pathological examination and had not received radiotherapy or chemotherapy prior to surgery. This study was approved by the Ethics Committee of The Second Affiliated Hospital of Wannan Medical College, and all participants provided informed consent prior to inclusion in the study.

### 2.3 Cell culture and treatment

A panel of CRC cell lines along with normal colonic epithelial cell lines (FHC) were procured from the American Type Culture Collection (Manassas, VA, USA). All cell lines were cultured in Dulbecco's modified Eagle's medium (DMEM) or RPMI‐1640 medium (Gibco BRL, Rockville, MD) supplemented with 100 μg/mL streptomycin, 100 U/mL penicillin and 10% fetal bovine serum (FBS; Gibco, NY, USA). The cultures were maintained at 37°C in a humidified atmosphere containing 5% CO_2_. To induce ferroptosis, the cells were treated with the ferroptosis agonist Erastin (10 μM, Selleck Chemicals, TX, USA) and then incubated for 24 h. For the inhibition of STAT3 activity, Caco2 and HT29 cells, stably expressing LV‐vec or LV‐ALMS1‐IT1, were treated with 10 μM stattic (Sigma‐Aldrich, S7947) for 2 days. For overexpress or knockdown of ALMS1‐IT1, HCT116, SW480, Caco2, and HT29 cells were selected for transfection experiments. pcDNA3.1‐ALMS1‐IT1 and si‐ALMS1‐IT1 (sequence: 5′‐TTGCGAAATAGTGTTTGTCTAGA‐3′) were obtained from GenePharma (Shanghai, China) for overexpression or knockdown of ALMS1‐IT1, vector were used as controls. Lipofectamine 2000 (Thermo Fisher Scientific, USA) facilitated transfection following the manufacturer's protocol.

### 2.4 Nuclear and cytoplasmic fraction isolation

Nuclear and cytoplasmic RNA extraction was conducted employing Thermo Fisher BioReagents (Thermo Fisher Scientific, Cat#AM1921) following the manufacturer's protocol. Initially, cells were suspended and lysed with cell fraction buffer, subsequently centrifuged at low speed to separate the nuclear fraction from the cytoplasmic fraction. The cytoplasmic fraction was carefully aspirated, leaving the nuclear pellet, which was then treated with cell disruption buffer. Finally, the samples were divided for RNA isolation using TRIzol (Thermo Fisher Scientific, Cat# 15596026).

### 2.5 Quantitative real‐time PCR (qRT‐PCR)

Total RNA was extracted from cells using the RNeasy Mini kit (Qiagen, USA) following the manufacturer's protocol. Complementary DNA (cDNA) was synthesized using a reverse‐transcribed assay with SYBR Premix Ex Taq II (TaKaRa, Shiga, Japan) according to the manufacturer's instructions. Subsequently, the expression levels of genes were assessed using qRT‐PCR analysis with SYBR Green I (Invitrogen), following the manufacturer's instructions. Data analysis was performed using the 2^−ΔΔCT^ method, with normalization to GAPDH. Primer sequences used were as follows: ALMS1‐IT1, F: 5′‐GCAGTGGTTCTTGACGGGTA‐3′, R: 5′‐CAGTCCAGCCTGGGCAATAA‐3′; CXCL9, F: 5′‐CCAGTAGTGAGAAAGGGTCGC‐3′, R: 5′‐AGGGCTTGGGGCAAATTGTT‐3′; CXCL10, F: 5′‐GTGGCATTCAAGGAGTACCTC‐3′, R: 5′‐TGATGGCCTTCGATTCTGGATT‐3′; TNFα, F: 5′‐CCTCTCTCTAATCAGCCCTCTG‐3′, R: 5′‐GAGGACCTGGGAGTAGATGAG‐3′; IFNα, F: 5′‐GCCTCGCCCTTTGCTTTACT‐3′, R: 5′‐CTGTGGGTCTCAGGGAGATCA‐3′; GAPDH, F: 5′‐GAAGGTGAAGGTCGGAGT‐3′, R: 5′‐GAGGATGGTGATGGGATTTC‐3′; U6, F: 5′‐CTCGCTTCGGCAGCACATA‐3′, R: 5′‐CGAATTTGCGTGTCATCCT‐3′. GAPDH and U6 were utilized as internal control.

### 2.6 Western blot

Briefly, cells were lysed using RIPA buffer supplemented with protease and phosphatase inhibitors. Protein concentration was determined using the BCA reagent (Beyotime, China). Subsequently, at least 20 μg of the protein sample was utilized for the detection of protein expression. The antibodies employed in the experiments included: anti‐pSTAT3 (1: 1000; CST, # 9145), anti‐STAT3 (1: 1000; CST, #9139), anti‐GAPDH (1:1000; CST, # 5174), SLC7A11 (1:1000, Abcam, #ab216876) anti‐GPX4 (1:1000, Abcam, #ab125066). Following protein transfer, the membranes underwent incubation with the corresponding secondary antibody (Abcam, 1:5000, ab7090) for 60 min, post‐washing with PBST. Visualization of the bands was accomplished using an ECL detection kit (Beyotime).

### 2.7 CCK‐8

Cell proliferation was assessed using the Cell Counting Kit‐8 (CCK‐8) from Dojindo, Japan. Briefly, CRC cells, both control and treated, were seeded onto 96‐well plates at an initial density of 2 × 10^3 cells per well. At the specified time points, 10 μL of CCK‐8 solution was added to each well. Following a 2‐h incubation period, the reaction products were quantified according to the manufacturer's instructions.

### 2.8 Colony formation

In the colony formation assay, around 500 treated cells were seeded into each medium and cultured for 7–14 days under conditions of 5% CO2 at 37°C with saturated humidity until colonies became visible to the naked eye. Following incubation, cells were fixed using 80% ethanol for 30 min, then the fixative was removed, and the cells were stained with 0.1% crystal violet for an additional 30 min. Subsequently, the number of colonies was enumerated.

### 2.9 Transwell

For the invasion assay, a transwell chamber coated with Matrigel matrix glue (100 μL; serum‐free medium diluted 1:6; BD Biosciences, San Jose, CA, USA) was employed. Cells were suspended in serum‐free culture medium, and approximately 1 × 10^5^ cells were added to the upper chamber. Simultaneously, the lower chamber was filled with complete medium serving as a chemoattractant. After 24 h of incubation, non‐invading cells were removed, and cells that had invaded through the membrane were fixed with 4% paraformaldehyde for 30 min, followed by staining with Diff‐Quik stain (Sysmex, Kobe, Japan) for an additional 30 min. After washing with PBS, the stained cells were counted.

The migration assay followed a similar protocol to the invasion assay, except for the absence of Matrigel matrix glue in the transwell chamber.

### 2.10 RNA pull‐down assay

The RNA pull‐down assay was conducted according to established protocols. Biotin‐labelled RNA probes were synthesized in vitro using the Pierce™ RNA 3′ End Biotinylation Kit (Thermo Fisher Scientific, USA) and the in vitro Transcription T7 Kit (TaKaRa, Japan), followed by purification with Trizol (TaKaRa, Japan). Each pull‐down experiment utilized 2 mg of purified biotinylated RNA. Cells were lysed in buffer containing 10 mM Tris–HCl (pH 7.4), 100 mM NaCl, 2.5 mM MgCl2, and 40 mg/mL digitalis saponins, followed by sonication and removal of cellular debris by centrifugation. The lysate was pre‐purified with streptavidin beads (Dyna‐beads M 280 streptavidin, Invitrogen, USA) and then incubated with the biotinylated RNA solution. After incubation, the RNA‐bound proteins were collected by overnight incubation with blocked beads. Wash steps were performed using high salt buffer (0.1% SDS, 1% Triton X‐100, 2 mM EDTA, 20 mM Tris–HCl, pH 8.0, 500 mM NaCl), low salt buffer (0.1% SDS, 1% Triton X‐100, 2 mM EDTA, 20 mM Tris–HCl, pH 8.0, 150 mM NaCl), and TE buffer. All buffers contained RNase, protease, and phosphatase inhibitors. The protein samples were eluted from the beads with SDS loading buffer and then subjected to analysis by either mass spectrometry or western blotting.

### 2.11 Mass spectrometry (LC–MS)

The mass spectrometry (LC–MS) analysis was performed using a Nano Aquity UPLC system (Waters Corporation, MA, US) coupled with a quadrupole Orbitrap mass spectrometer (Q‐Exactive) (Thermo Fisher Scientific, Bremen) equipped with an online nanoelectrospray ion source. Peptide samples were prepared by dissolving them in 10 μL of solvent A (5% acetonitrile, 0.1% formic acid in water) and injected onto a Thermo Scientific Acclaim PepMap C18 column (100 μm × 2 cm, 5 μm, Thermo Fisher Scientific) at a flow rate of 10 μL/min for 3 min. Following this, peptides were separated on an analytical column (Acclaim PepMap C18, 75 μm × 15 cm, 2 μm, 100 Å) using a linear gradient starting from 2% solvent B (90% acetonitrile, 0.1% formic acid in water) and increasing to 45% B over 75 min, followed by re‐equilibration at initial conditions for 15 min. The column flow rate was maintained at 300 nL/min, and the column temperature was set to 40°C. An electrospray voltage of 2.2 kV relative to the mass spectrometer inlet was applied. MS raw files generated by the Q‐Exactive instrument were analysed using MaxQuant software (version 1.5.2.8, http://www.maxquant.org/) for protein identification and quantification. The data were searched against the Human UniProtKB/Swiss‐Prot database using the Andromeda search engine. Parameters included a minimum peptide length of seven amino acids, trypsin cleavage specificity with allowance for up to two missed cleavages, initial mass deviation of precursor and fragment ions set to 10 ppm and 0.5 Da, respectively, false discovery rate (FDR) at both peptide and protein levels set to 1%, and a minimum peptide segment length of 5.

### 2.12 RNA immunoprecipitation (RIP)

Initially, wild‐type cells from three 10 cm plates underwent fixation using 1% formaldehyde in PBS for 10 min at room temperature, followed by quenching with 100 mM glycine for 5 min. After three washes with pre‐cooled PBS, the cells were suspended in 500 μL of RIP lysis buffer (1% SDS, 10 mM EDTA, 50 mM Tris–HCl pH 8.1), supplemented with protease inhibitor cocktail and RNAase inhibitor, and rotated for 30 min at 4°C. Subsequently, the lysate was sonicated and centrifuged at 4°C, 12000 rpm for 10 min to remove debris. The resulting supernatant was collected, and RIP buffer (0.01% SDS, 1.1% Triton X‐100, 1.2 mM EDTA, 16.7 mM Tris–HCl pH 8.1, 167 mM NaCl) was added to reach a final volume of 1 mL. Then, 50 μL of pre‐washed Protein A/G Agarose beads (Thermo Fisher Scientific, USA) were added and incubated at 4°C with rotation for 1 h to pre‐clear the lysate. Following pre‐clearing, the mixture was centrifuged at 10000 rpm for 5 min, and the supernatant was transferred. A portion of 100 μL was kept as input, while the remainder was divided into two equal parts for IgG and IP (Anti‐STAT3) groups, respectively. Incubation was carried out overnight at 4°C with rotation. On the following day, pre‐washed agarose beads were added, and the mixture was incubated with rotation at 4°C for 2 h. Afterward, the beads were washed three times with RIP buffer, followed by the addition of 500 μL Trizol (TaKaRa, Japan) to each sample for RNA isolation, following the manufacturer's instructions.

### 2.13 Fluorescence in situ hybridization (FISH)

FISH experiments were carried out following the manufacturer's protocol from RiboBio (Guangzhou, China). In brief, CRC cells were seeded into a 35 mm glass‐bottom dish (NEST, Jiangsu, China) and incubated overnight in complete medium. Following this, cells were fixed with 4% paraformaldehyde (Solarbio, Beijing, China) for 10 min at room temperature, permeabilized with 0.5% Triton X‐100 (Amresco) for 5 min at 4°C, and blocked with pre‐hybridization buffer (ZSJB‐BIO, Beijing, China) for 30 min at 37°C. After blocking, cells were incubated with 40 nM FISH probes specific to ALMS1‐IT1 and STAT3 (RiboBio, Guangzhou, China) overnight at 37°C in the dark. Subsequently, cells underwent washing steps with hybridization washing buffers I (4 × SSC, 0.1% Tween‐20), II (2 × SSC), and III (1 × SSC) successively at 42°C in the dark. Nuclei were counterstained with DAPI (0.1 g/mL), followed by three washes with PBS. Images were captured using a Carl Zeiss laser confocal microscope, where the subcellular localization of ALMS1‐IT1 and STAT3 was determined by the distribution of red and green signals.

### 2.14 Detection of GSH/GSSG, ROS and lipid peroxidation

The levels of glutathione (GSH) and glutathione disulfide (GSSG) were determined using the GSH and GSSG test kit (Beyotime, China) following the manufacturer's instructions. Similarly, the assessment of ROS levels was conducted using a commercial kit (Beyotime, China) according to the manufacturer's protocol. Briefly, cells were pre‐incubated with 2′,7′‐dichlorofluorescin diacetate (DCFH‐DA) at 37°C for 30 min. After removal of the extracellular dye, the cells were washed three times and then incubated with serum‐free DMEM. Subsequently, fluorescence was measured using a microplate reader (Thermo Fisher Scientific, USA) with excitation at 488 nm and emission at 525 nm. The relative concentration of malondialdehyde (MDA), indicative of lipid peroxidation, in cell lysates was assessed using a Lipid Peroxidation (MDA) Assay Kit (Abnova, China) as per the manufacturer's instructions.

### 2.15 Statistical analysis

Statistical analysis was performed on data obtained from three independent experiments using SPSS 19.0 (IBM, Armonk, NY). Results are presented as means ± standard deviation (SD). Student's *t*‐tests were used to analyse differences between two groups, while one‐way analysis of variance (ANOVA) was applied for comparisons involving multiple groups. A significance threshold of *p* < 0.05 was considered statistically significant.

## 3 RESULTS

### 3.1 Identifying ALMS1‐IT1 as a significant upregulated ferroptosis‐related gene in CRC

**FIGURE 1:**
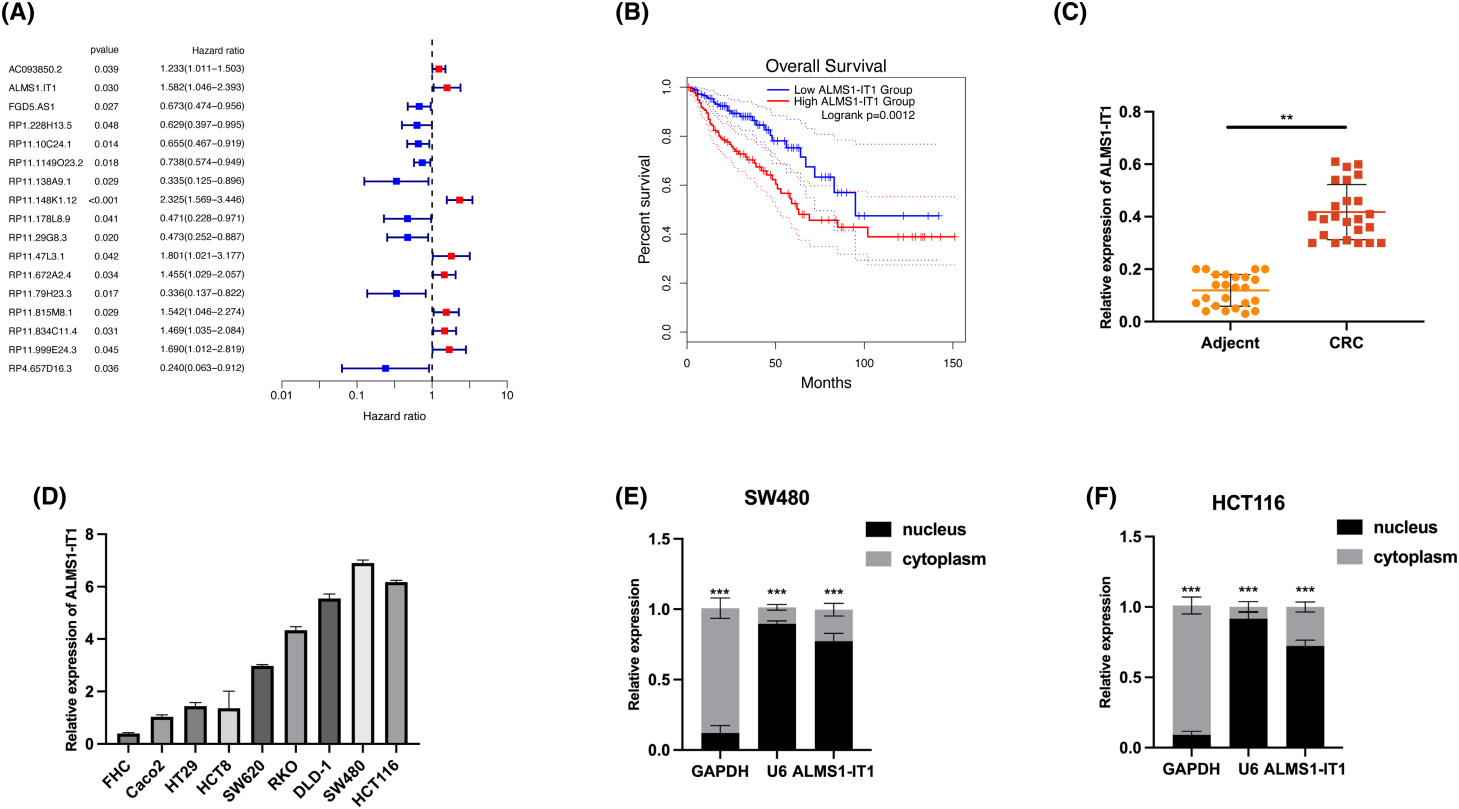
Identifying ALMS1‐IT1 as a significant upregulated ferroptosis‐related gene in CRC. (A) The results of the univariate COX analysis of ferroptosis‐related lncRNAs in CRC tissues were analysed based on the transcriptome data in the TCGA database (https://portal.gdc.cancer.gov/). (B) Kaplan–Meier survival analysis results of ALMS1‐ IT1 in CRC samples. (C) Measurement of ALMS1‐ IT1 expression in 24 pairs of CRC clinical adjacent and tumour tissues. (D) Measurement of ALMS1‐ IT1 expression in CRC tumour cell lines and FHC (normal colonic epithelial cells). (E, F) Subcellular distribution assay results of ALMS1‐ IT1 in the nucleus and cytoplasm of HCT116 and SW480 cells. Data shown represent mean ± SD from three independent experiments. **p < 0.01, ***p < 0.001.

Ferroptosis, characterized as a regulated form of cell demise (RCD), distinguishes itself from alternative modes of cellular demise through distinct attributes encompassing morphology, biochemical features and genetic mechanisms.^13^ A growing body of contemporary research indicates that the induction of ferroptosis in CRC cells can hold therapeutic potential in clinical treatment.^18^, ^19^ Our investigation aimed to elucidate the role of ferroptosis‐related lncRNAs in CRC progression. Through bioinformatic analysis of public high‐throughput data, we identified the top 17 most dysregulated ferroptosis‐related lncRNAs in CRC (Figure 1A). Among these, the lncRNA ALMS1‐IT1 showed a significant correlation with overall survival in CRC patients; specifically, high expression levels of ALMS1‐IT1 were associated with poorer prognoses (Figure 1B). Further analysis of 24 pairs of CRC samples confirmed a substantial upregulation of ALMS1‐IT1 in CRC tumour tissues compared to adjacent normal tissues (Figure 1C). This expression pattern was also observed in CRC tumour cell lines in comparison to the normal cell line FHC (Figure 1D). Notably, subcellular localization analysis indicated that ALMS1‐IT1 is predominantly localized in the nucleus (Figure 1E,F), suggesting its potential functional relevance in CRC progression.

### 3.2 The promotive effect of ALMS1‐IT1 knockdown on the ferroptotic activity in CRC cells

**FIGURE 2:**
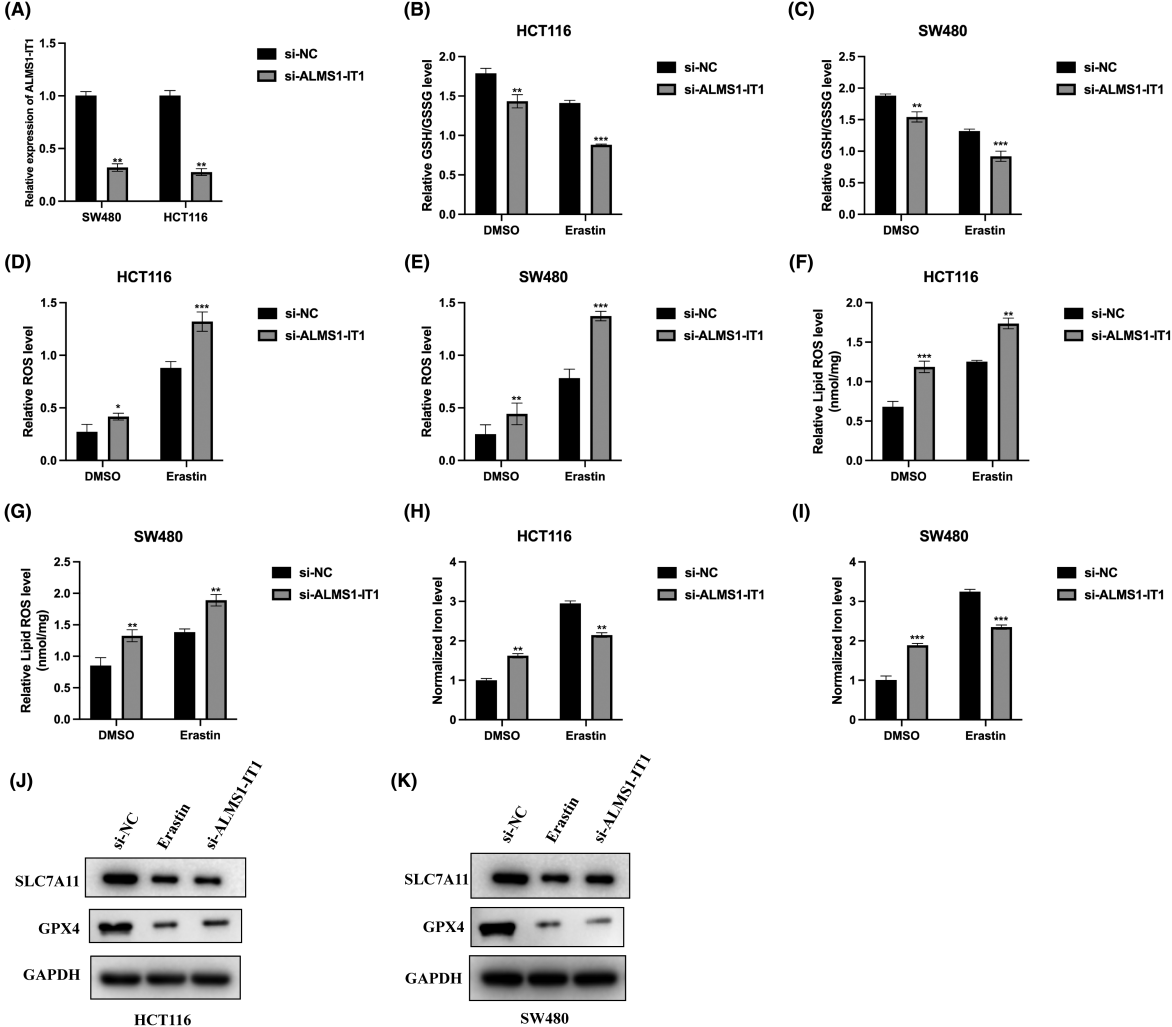
The promotive effect of ALMS1‐IT1 knockdown on the ferroptotic activity in CRC cells. (A) ALMS1‐IT1 knockdown cell models were generated by stably introducing si‐NC and si‐ALMS1‐IT1 into HCT116 and SW480 cells, results were verified by qRT‐PCR. GSH/GSSG ratios (B, C), ROS levels (D, E), lipid ROS (F, G), and Fe2+ concentration (H, I) was measured in the four indicated cell lines after treated with 10 μM erastin for 48 h. (J, K) Ferroptosis markers (SLC7A11 and GPX4) in CRC cells with or without erastin/si‐ALMS1‐IT1 treatment were detected by western blot. Data shown represent mean ± SD from three independent experiments. *p < 0.05, **p < 0.01, ***p < 0.001.

Since ALMS1‐IT1 was identified as a ferroptosis‐related lncRNA in CRC tissues, the effect of ALMS1‐IT1 in CRC cells were investigated. ALMS1‐IT1 knockdown cell models were constructed for experimental application and were verified by qRT‐PCR (Figure 2A). Ferroptosis entails the accrual of iron‐dependent lipid peroxides (lipid‐ROS), ultimately leading to cellular demise.^13^ This process is categorized into upstream events, involving the production of ROS, and downstream mechanisms responsible for executing ferroptotic cell death.^20^ In our investigation, we induced ferroptosis through the administration of erastin in HCT‐116 and 480 cell lines. Given the crucial involvement of glutathione (GSH)/glutathione disulfide (GSSG), ROS, lipid ROS, and ferrous iron (Fe^2+^) in the ferroptotic pathway, we subsequently quantified their levels in erastin‐treated CRC cells. Our findings demonstrated that the knockdown of ALMS1‐IT1 heightened the susceptibility of cells to ferroptosis. This was evident from the decreased levels of GSH/GSSG (Figure 2B,C), alongside increased accumulation of ROS (Figure 2D,E), lipid ROS (Figure 2F,G) and Fe^2+^ (Figure 2H,I) following erastin induction. Furthermore, we observed a reduction in the expression levels of ferroptosis markers (SLC7A11 and GPX4) in erastin‐treated CRC cells, and a similar trend was observed at the protein level in ALMS1‐IT1 knockdown CRC cells (Figure 2J,K). These results underscore the augmentative impact of ALMS1‐IT1 knockdown on ferroptotic activity in CRC cells.

### 3.3 ALMS1‐IT1 knockdown inhibits proliferation and metastasis of CRC

**FIGURE 3:**
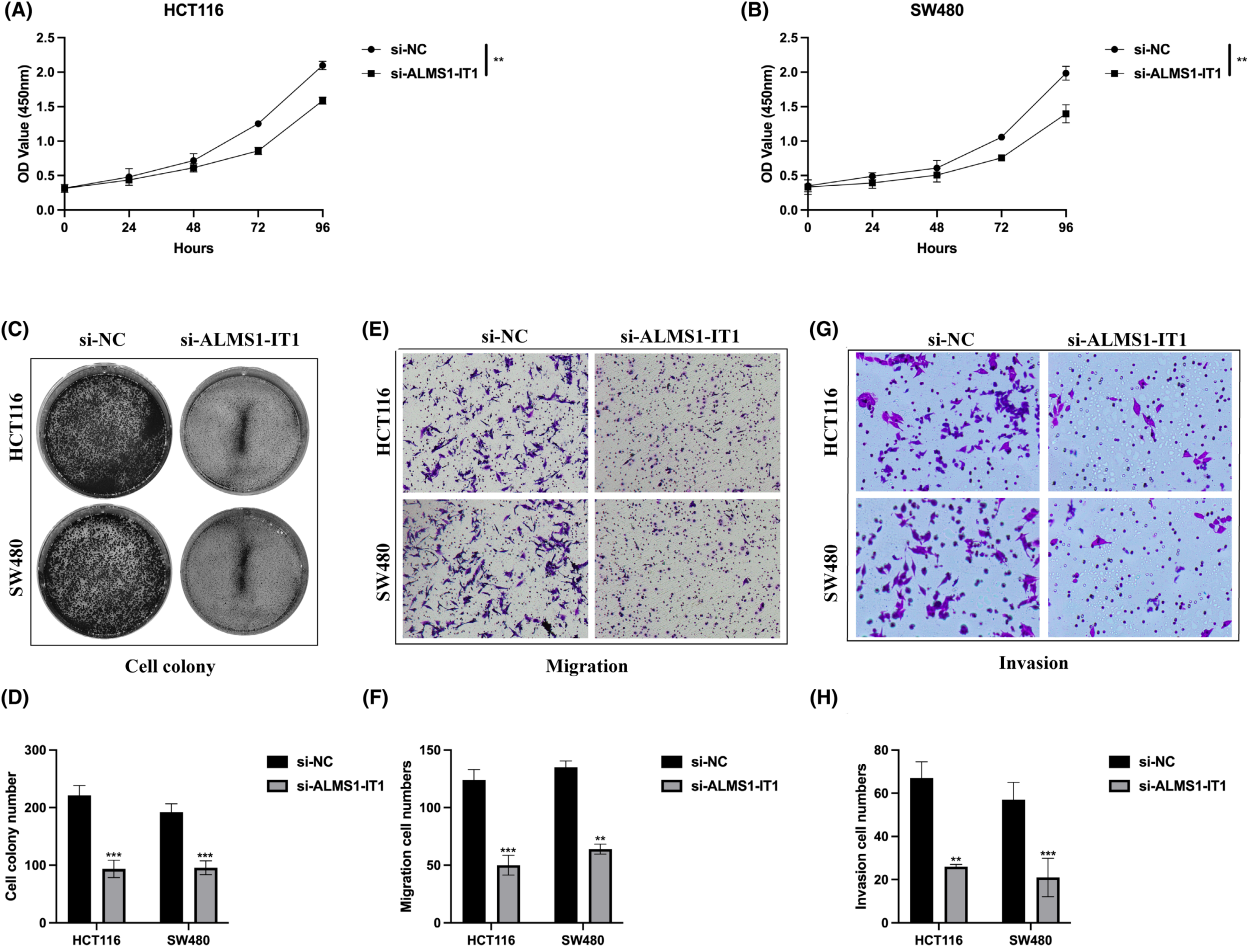
ALMS1‐IT1knockdown inhibits proliferation and metastasis of CRC. (A, B) Cell viability was evaluated by CCK‐8 assay. (C, D) Cell clonogenic ability was detected by performing colony formation assay. (E, F) Cell migration capability was measured by conducting transwell migration assay. (G, H) Cell ivasion level was demonstrated by utilizing transwell invasion assay. Data shown represent mean ± SD from three independent experiments. **p < 0.01, ***p < 0.001.

Subsequently, we investigated the functional significance of ALMS1‐IT1 in CRC cellular behaviours. Using CCK‐8 and cell colony formation assays, we found that knockdown of ALMS1‐IT1 led to a reduction in both cell viability (Figure 3A,B) and clonogenic potential (Figure 3C,D). Additionally, we assessed the impact of ALMS1‐IT1 on the metastatic capabilities of CRC cells. Results from transwell migration and invasion assays demonstrated that ALMS1‐IT1 knockdown resulted in a decreased ability of CRC cells to migrate (Figure 3E,F) and invade (Figure 3G,H). Collectively, these findings suggest that ALMS1‐IT1 functions as an oncogene, contributing to CRC progression.

### 3.4 ALMS1‐IT1interacts and activates STAT3

**FIGURE 4:**
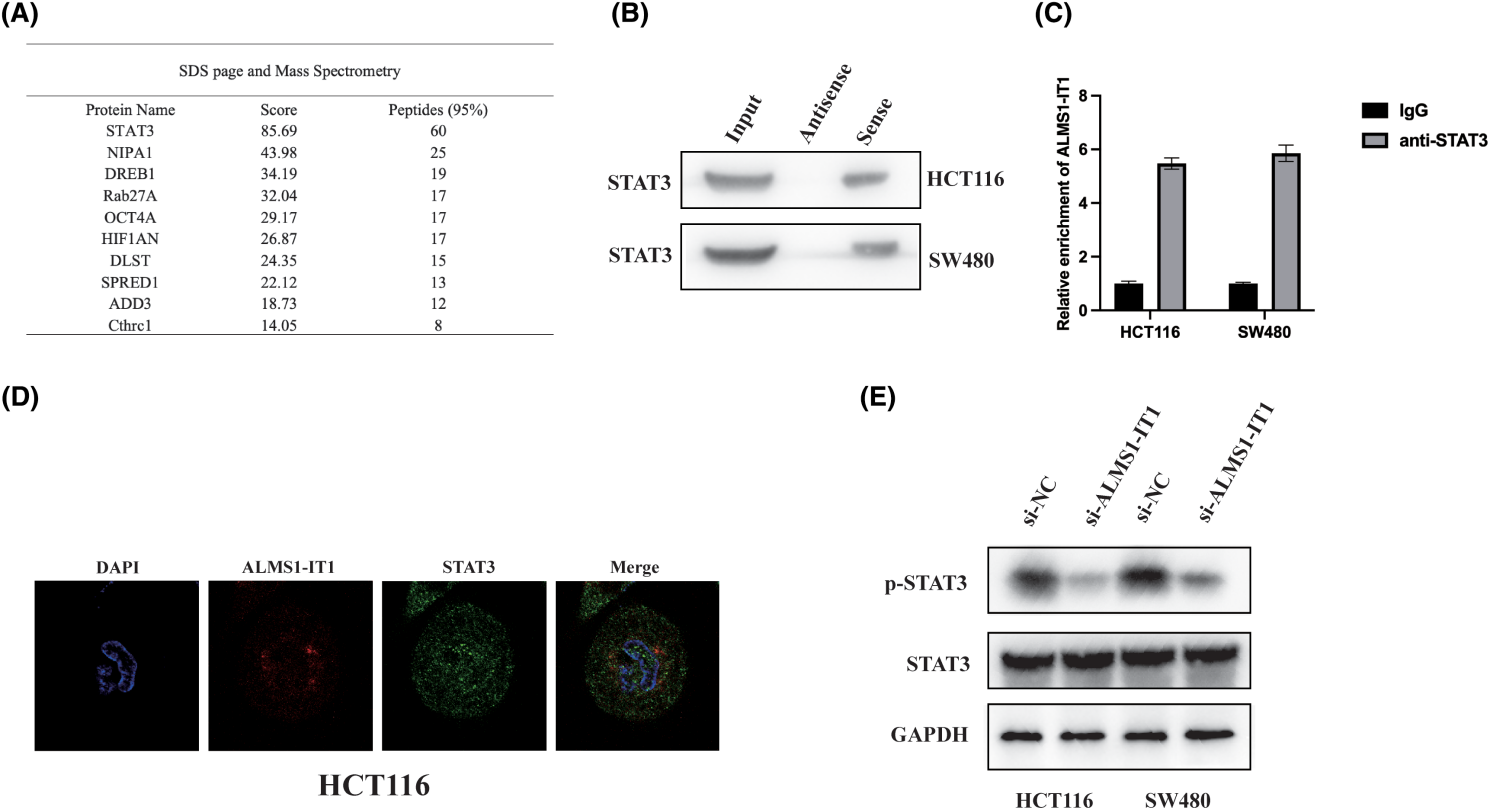
ALMS1‐IT1 interacts and activates STAT3. (A) RNA pull‐down coupled with mass spectrometry analysis was conducted to elucidate the repertoire of proteins that interact with ALMS1‐IT1. Biotin‐labelled ALMS1‐IT1 was incubated with lysates derived from HCT116 cells to facilitate the pull‐down of ALMS1‐IT1‐associated proteins. Subsequently, a specific fragment was isolated and subjected to mass spectrometry analysis. The top 10 candidate proteins identified in this analysis are enumerated. (B) Western blot analysis was conducted on proteins extracted from both antisense ALMS1‐IT1 and ALMS1‐IT1 pull‐down assays. (C) RIP experiments were carried out using an anti‐STAT3 antibody, followed by the utilization of specific primers to detect ALMS1‐IT1. (D) Immunofluorescence in situ hybridization (ImmunoFISH) images depicting the colocalization of ALMS1‐IT1 and STAT3 in HCT116 cells. (E) Western blot analysis was performed to assess the levels of phosphorylated STAT3 (p‐STAT3) and total STAT3 in HCT116 and SW480 cells following si‐ALMS1‐IT1.

Due to the potential for lncRNAs to exert their functions through interactions with cellular proteins, we posited that ALMS1‐IT1 might engage in interactions with specific proteins to modulate biological processes. To elucidate the mechanistic underpinnings of ALMS1‐IT1 in regulating ferroptosis and CRC tumorigenic properties, we initially employed RNA pulldown assays coupled with mass spectrometry analysis. This approach enabled the identification of a ALMS1‐IT1‐protein complex in cell lysates obtained from HCT116 cells, with the antisense of ALMS1‐IT1 serving as a negative control in these experiments (Figure 4A). Among the plethora of proteins identified, signal transducer and activator of transcription 3 (STAT3) emerged as particularly intriguing. We corroborated the presence of STAT3 within an intact complex through independent RNA pulldown assays conducted in HCT116 and SW480 cells (Figure 4B). Furthermore, RNA immunoprecipitation (RIP) assays validated the enrichment of ALMS1‐IT1 in the complexes precipitated with the antibody against STAT3 in HCT116 and SW480 cells (Figure 4C). Additionally, fluorescence in situ hybridization (FISH) staining depicted colocalization between ALMS1‐IT1 and STAT3 (Figure 4D). Of note, we observed that the ALMS1‐IT1 knockdown resulted in a decrease in the levels of phosphorylated STAT3 (p‐STAT3), while the overall levels of STAT3 remained unaffected (Figure 4E). These findings suggest that ALMS1‐IT1 may exert control over STAT3 phosphorylation.

### 3.5 ALMS1‐IT1 aggravates immune evasion in CRC via activating STAT3

**FIGURE 5:**
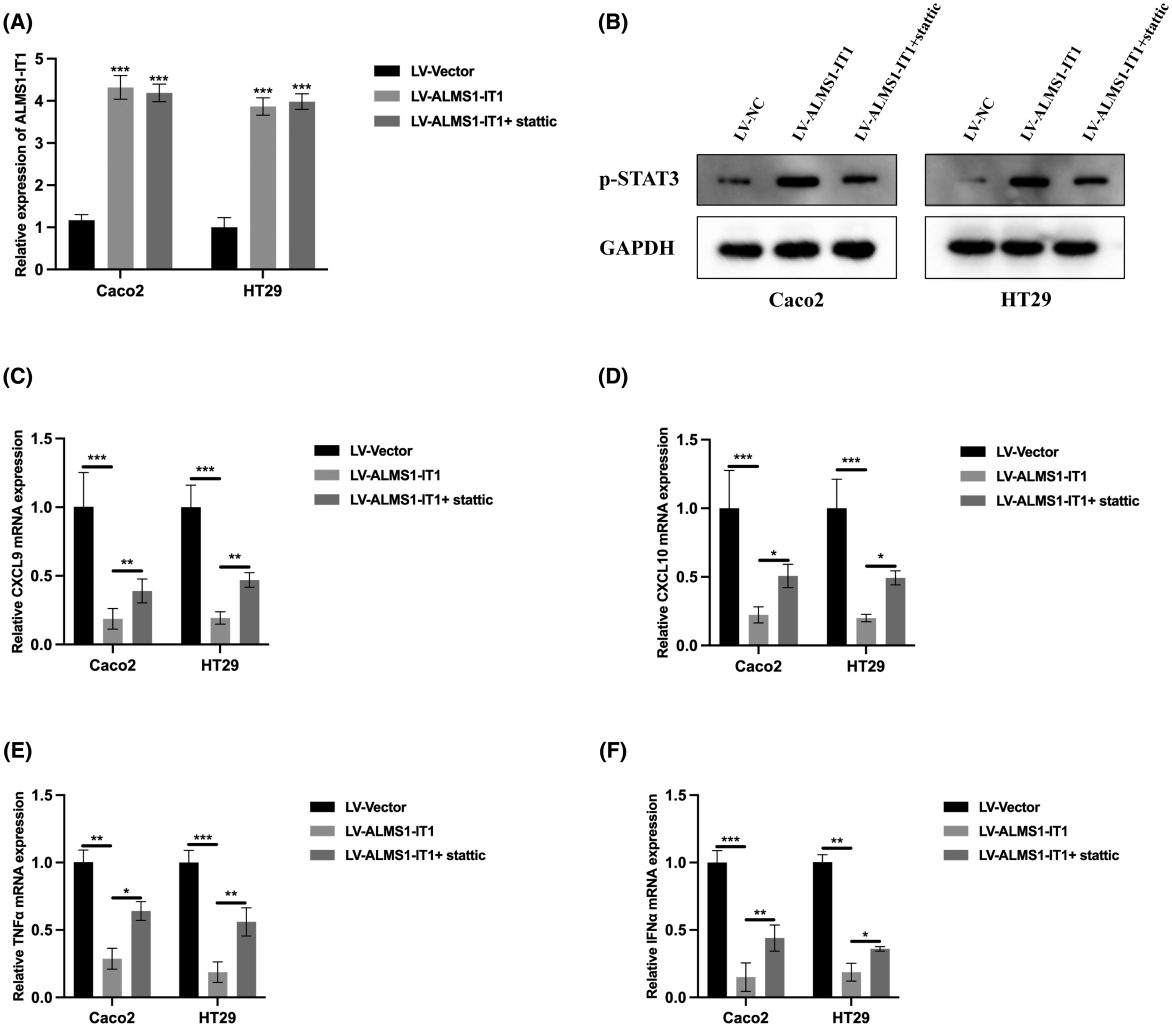
ALMS1‐IT1 aggravates immune evasion in CRC via activating STAT3. (A) ALMS1‐IT1 overexpression cell models were constructed and the expression level of ALMS1‐IT1 was verified by qRT‐PCR. (B) The expression of p‐STAT3 in ALMS1‐IT1 overexpression cell models were measured by western blot. (C–F) Relative mRNA levels of inflammatory mediators after ALMS1‐IT1 overexpression or combined with STAT3 phosphorylation inhibitor stattic treatment were detected by qRT‐PCR. Data shown represent mean ± SD from three independent experiments. *p < 0.05, **p < 0.01, ***p < 0.001.

A previous study has documented that interferon alpha (IFNα)‐activated STAT3 dampens the induction of inflammatory mediators such as the chemokines CXCL9 and CXCL10.^21^ Additionally, another investigation revealed that p‐STAT3 attenuates the type I interferon (IFN) response.^22^ To elucidate whether ALMS1‐IT1 modulates CRC immune response via STAT3, we generated ALMS1‐IT1 overexpression cell models by introducing ALMS1‐IT1 plasmids into Caco2 and HT29 cells, transfection effciencies were verified by qRT‐PCR and western blot (Figure 5A,B). The results obtained from RT‐qPCR analysis revealed that the upregulation of ALMS1‐IT1 led to an decrease in the expression levels of various chemokines and proinflammatory factors, including CXCL9, CXCL10, TNFα and IFNα (Figure 5C–F). Conversely, the overexpression of ALMS1‐IT1 combined with stattic (a specific STAT3 phosphorylation inhibitor) exhibited a contrasting effect, resulting in increased expression levels of these factors (Figure 5C–F). Our results indicated that ALMS1‐IT1 promotes CRC immune evasion phenomena via STAT3.

### 3.6 STAT3 activation is responsible for the inhibitive effect of ALMS1‐IT1 on ferroptosis activity

**FIGURE 6:**
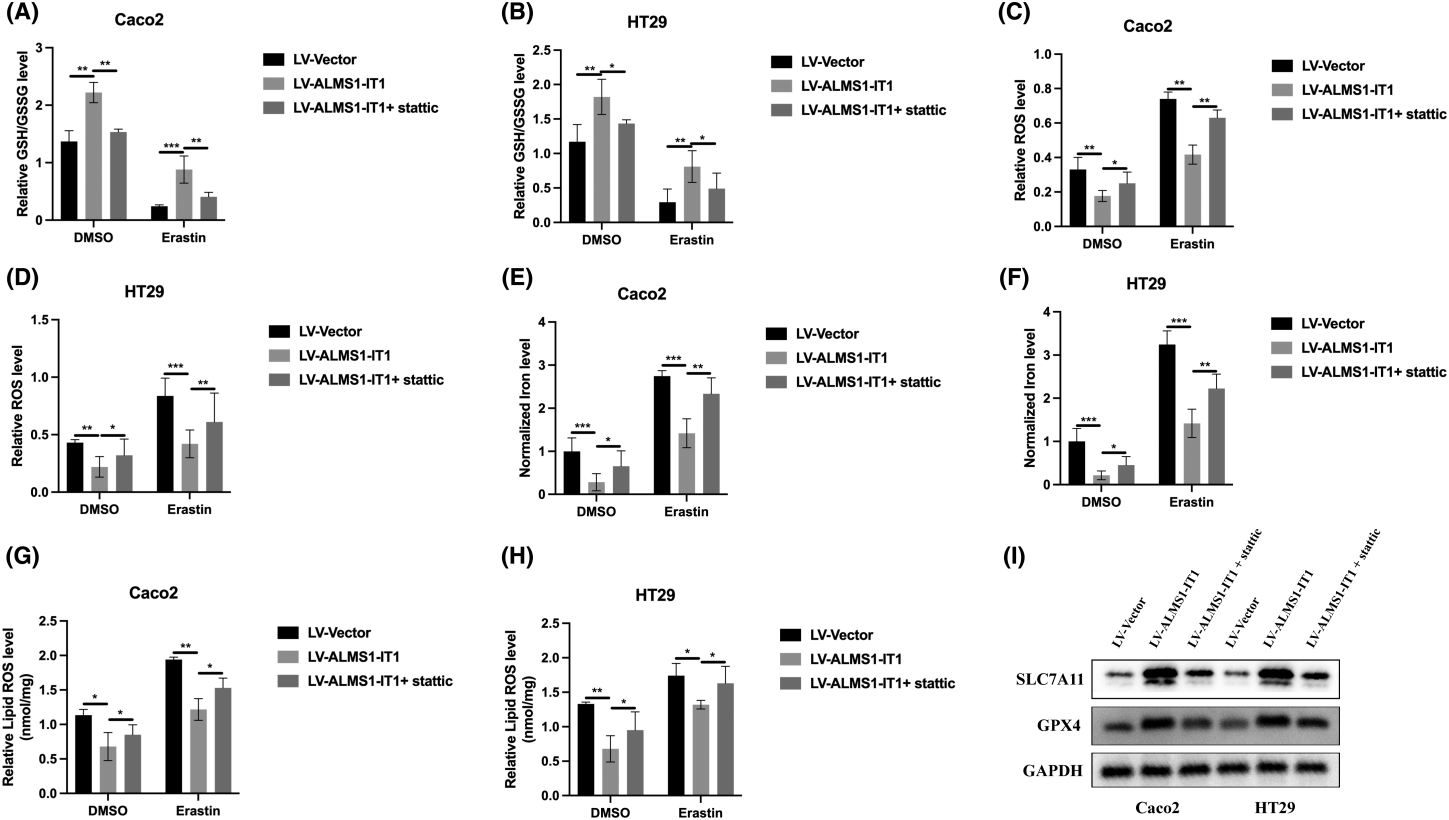
STAT3 activation is responsible for the inhibitive effect of ALMS1‐IT1 on ferroptosis activity. GSH/GSSG ratios (A, B), ROS levels (C, D), Lipid ROS (E, F), and Fe^2+^ concentration (G, H) was measured in the indicated cell lines after treated with 10 μM erastin for 48 h. (I) Ferroptosis markers (SLC7A11 and GPX4) in CRC cell models were detected by western blot. Data shown represent mean ± SD from three independent experiments. *p < 0.05, **p < 0.01, ***p < 0.001.

To elucidate whether ALMS1‐IT1 modulates ferroptosis activity in CRC by promoting STAT3 phosphorylation, we conducted experiments using cell models overexpressing ALMS1‐IT1 and treated them with a combination of ALMS1‐IT1 overexpression and stattic, a STAT3 inhibitor. The overexpression of ALMS1‐IT1 was found to decrease the susceptibility of CRC cells to ferroptosis. This was evidenced by increased levels of GSH/GSSG (Figure 6A,B) and a concomitant decrease in the accumulation of ROS (Figure 6C,D), lipid ROS (Figure 6E,F) and Fe^2+^ (Figure 6G,H). Additionally, ALMS1‐IT1 overexpression resulted in upregulation of ferroptosis markers, specifically SLC7A11 and GPX4 (Figure 6I). Conversely, treatment with both ALMS1‐IT1 overexpression and stattic reversed these effects (Figure 6A–I). These findings collectively suggest that ALMS1‐IT1 inhibits ferroptosis by promoting STAT3 phosphorylation in CRC cells.

### 3.7 ALMS1‐IT1 promotes CRC progression through activating STAT3

**FIGURE 7:**
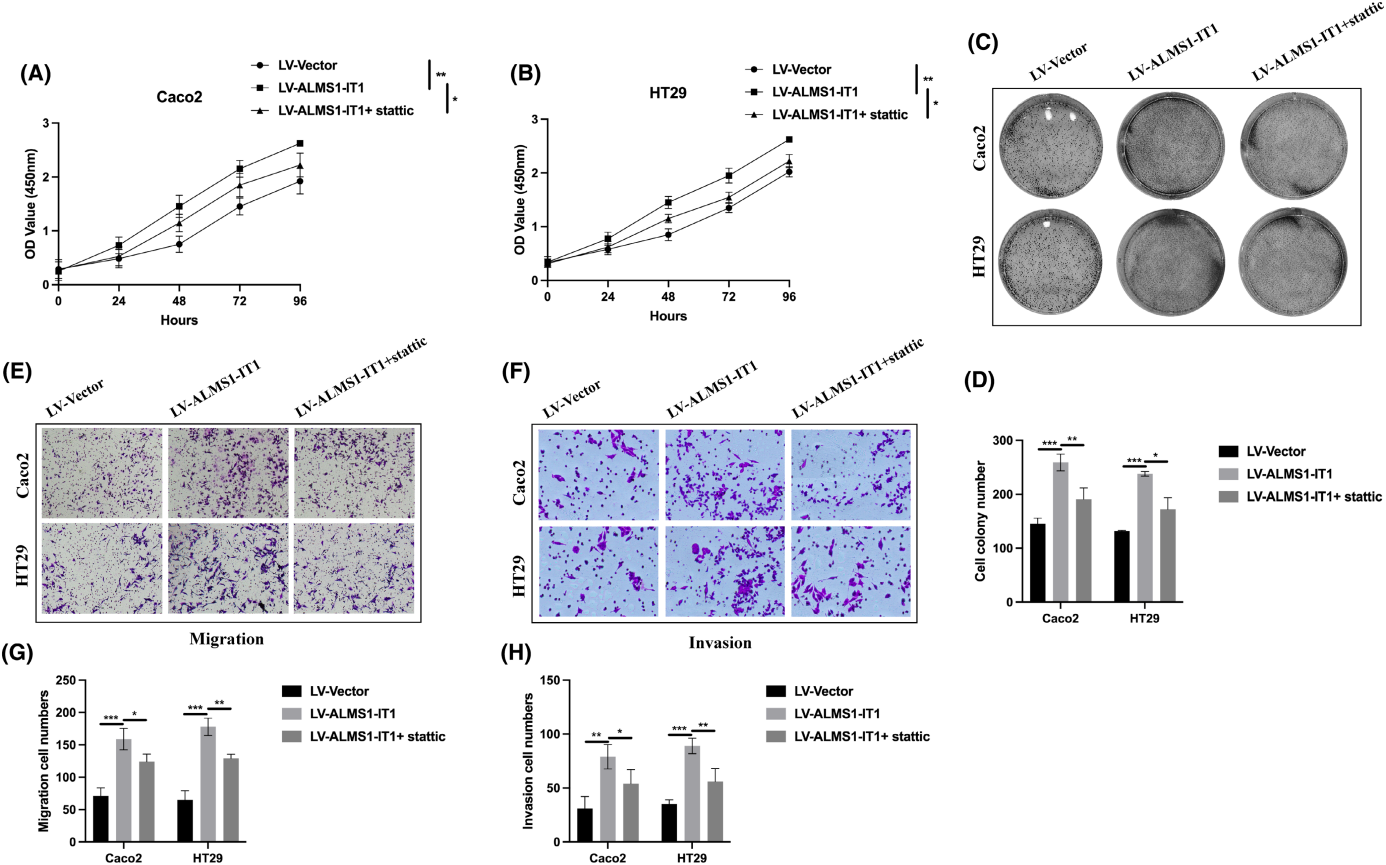
ALMS1‐IT1 promotes CRC progression through activating STAT3. (A, B) Cell viability was assessed using the CCK‐8 assay. (C, D) The clonogenic ability of cells was determined through colony formation assays. (E–G) Cell migration capacity was evaluated via transwell migration assays. (F–H) The invasive potential of cells was demonstrated using transwell invasion assays. Data shown represent mean ± SD from three independent experiments. *p < 0.05, **p < 0.01, ***p < 0.001.

Subsequently, we sought to elucidate the impact of ALMS1‐IT1/STAT3 on the tumorigenic properties of CRC. Utilizing CCK‐8 and cell colony formation assays, we observed that overexpression of ALMS1‐IT1 resulted in an increase in both cell viability (Figure 7A,B) and clonogenic potential (Figure 7C,D). Furthermore, we investigated the influence of ALMS1‐IT1 on the metastatic capabilities of CRC cells. Results from transwell migration and invasion assays revealed that ALMS1‐IT1 overexpression enhanced the migratory (Figure 7E–G) and invasive (Figure 7F–H) abilities of CRC cells. However, these exacerbated effects of ALMS1‐IT1 overexpression on CRC cellular behaviours were attenuated by stattic treatment (Figure 7A–H). Collectively, our findings suggest that ALMS1‐IT1 functions as an oncogene, contributing to CRC progression through STAT3 phosphorylation.

## 4 DISCUSSION

The official designation of ALMS1‐IT1 is ALMS1 intronic transcript 1. As of the current understanding, research on ALMS1‐IT1 remains limited, yet certain studies posit that it harbours prognostic significance.^23^ Recent scholarly work has postulated a potential association between the expression of ALMS1‐IT1 and ferroptosis.^24^ Through bioinformatics analyses, predictions suggest ALMS1‐IT1's utility as a prognostic biomarker for Head and Neck Squamous Cell Carcinoma (HNSCC).^25^, ^26^ Experimental evidence indicates that the interaction between ALMS1‐IT1 and AVL9 may contribute to the malignant advancement of Lung Adenocarcinoma (LUAD) by influencing the cyclin‐dependent kinase pathway.^27^ Drawing upon extant scholarly findings, it is hypothesized that ALMS1‐IT1 could play a pivotal role in the pathogenesis and progression of CRC. Through the integration of bioinformatics analysis and clinical sample assessments, it has been discerned that ALMS1‐IT1 is implicated not merely in relation to ferroptosis but also exhibits a significant correlation with the overall survival rate of CRC patients. Elevated expression levels of ALMS1‐IT1 are indicative of an adverse prognosis for individuals diagnosed with CRC, suggesting that ALMS1‐IT1 may function as an oncogenic factor in the progression of CRC by modulating ferroptosis activity.

Ferroptosis is characterized as a RCD mechanism driven by the iron‐catalysed accumulation of lipid peroxides. This process is influenced by several factors, including the attenuation of glutathione peroxidase 4 (GPX4) functionality, a deficiency in cysteine, and the peroxidation of arachidonic acid (AA).^28^, ^29^, ^30^ Three preclinical findings substantiate a connection between specific carcinogenic signals and the induction of ferroptosis: First, the discovery of erastin as a ferroptosis activator emerged from its unique capability to selectively induce cell death in cancer cells with mutant RAS, sparing those with wild‐type RAS.^31^ Second, the RAS–RAF–MEK–ERK signalling cascade must be activated for erastin to exert its lethal effect on these cells.^32^ Third, iron, known for its vital role in cancer cell proliferation, is also indispensable for the cell death triggered by erastin.^13^ A burgeoning corpus of research underscores that provoking ferroptosis—by amplifying intracellular Fe^2+^ concentrations, escalating levels of ROS, curtailing the availability of the antioxidant glutathione (GSH), or rendering GPX4 inactive—can significantly influence the therapeutic landscape of CRC.^18^, ^19^ In contrast, thwarting ferroptosis may engender tumour progression and engender resistance to established therapeutic regimens in CRC.^33^ Given this research background, we thoroughly examined the role of ALMS1‐IT1 in modulating ferroptosis in CRC cells. Our findings revealed that reduced ALMS1‐IT1 expression markedly heightened the cells' susceptibility to ferroptosis. This was demonstrated by a significant decrease in GSH/GSSG ratios and a notable increase in the accumulation of lipid‐based ROS and Fe^2+^ following erastin treatment. Furthermore, we observed a substantial downregulation of key ferroptosis markers, SLC7A11 and GPX4, both at the mRNA and protein levels in CRC cells with ALMS1‐IT1 knockdown. These results underscore ALMS1‐IT1's pivotal role in regulating ferroptosis and suggest its potential as a therapeutic target in CRC.

Due to the markedly effect of ALMS1‐IT1 on ferroptosis activity in CRC cells, following experiments were focused on tumorigenic properties of CRC cells. Results of CCK‐8, colony formation, and transwell assays indicated that ALMS1‐IT1 promoted CRC proliferation and metastasis. In addition, we also investigated the underlying molecular mechanisms of ALMS1‐IT1. We aimed to a interact protein with ALMS1‐IT1 in CRC cells. By utilizing serial experiments, STAT3 was identified as an associate protein. STAT3 functions as a critical downstream effector of the JAK–STAT signalling cascade, which plays a pivotal role in mediating the inflammatory immune response.^34^ Dysregulated activation of STAT3 has been documented across a spectrum of human solid malignancies, encompassing CRC.^35^ Furthermore, STAT3 facilitates the establishment of an immunosuppressive milieu through the modulation of immune factor expression and the recruitment of immunosuppressive cellular elements.^36^ Our study revealed that ALMS1‐IT1 directly interacts with STAT3 in CRC cells, leading to an increase in STAT3 phosphorylation levels. This suggests the potential for ALMS1‐IT1 to modulate immune evasion in CRC cells through STAT3 activation. Subsequent experiments confirmed this hypothesis. Furthermore, rescue experiments provided additional evidence that STAT3 activation is crucial for the effects of ALMS1‐IT1 on both ferroptosis and tumorigenesis.

While our study has demonstrated the significant role of ALMS1‐IT1 in ferroptosis, tumorigenesis, and immune evasion activities in CRC, and has partially elucidated its underlying molecular mechanisms, further research is necessary to fully understand its effects. Future studies should aim to: (1) Verify the role of ALMS1‐IT1 in ferroptosis, tumorigenesis, and immune evasion using animal models. (2) Elucidate the specific mechanisms underlying the interaction between ALMS1‐IT1 and STAT3. (3) Assess the clinical significance of ALMS1‐IT1 in CRC by analysing a larger cohort of human samples. These additional studies will provide a more comprehensive understanding of ALMS1‐IT1's role in CRC and its potential as a therapeutic target.

In summary, our study identified ALMS1‐IT1 as significantly upregulated in CRC tissues, with a strong correlation to poor prognosis. ALMS1‐IT1 was found to suppress ferroptosis and promote oncogenic activities such as cell proliferation, migration, and invasion in CRC. It operates by interacting with and activating STAT3, thereby enhancing immune evasion and influencing both ferroptosis and tumorigenesis. These findings suggest that the ALMS1‐IT1‐STAT3 axis represents a novel therapeutic target in CRC, providing insights into potential interventions aimed at disrupting this pathway to inhibit tumour progression and improve patient outcomes.

## AUTHOR CONTRIBUTIONS

**Zhaoying Wu:** Data curation (equal); formal analysis (equal); investigation (equal); methodology (equal); software (equal); validation (equal); visualization (equal). **Junwei Zou:** Data curation (equal); investigation (equal); software (equal); visualization (equal). **Hao Xie:** Methodology (equal); software (equal); validation (equal); visualization (equal). **Jie Wang:** Data curation (equal); validation (equal). **Yong Huang:** Software (equal); visualization (equal). **Fei Liu:** Conceptualization (equal); investigation (equal); resources (equal); supervision (equal). **Chungen Xing:** Conceptualization (equal); funding acquisition (equal); investigation (equal); project administration (equal); resources (equal); supervision (equal); validation (equal); writing – original draft (equal).

## FUNDING INFORMATION

This work was supported by Health research project of Anhui Province (Grant/Award Number: AHWJ2023A30148); The Key project of Natural Science Foundation of Wannan Medical College (Grant/Award Number: WK2023ZZD38); The Climbing Scientific Peak Project for Talents, the Second Affiliated Hospital of Wannan Medical College (Grant/Award Number: DFJH2022016); Pre‐research fund of the Second Affiliated Hospital of Soochow University (Grant/Award Number: SDFEYLC2345).

## CONFLICT OF INTEREST STATEMENT

The authors declare that they have no conflict of interest.

## Data Availability

Data will be made available on request.
